# Nanoinhibitory Impacts of Salicylic Acid, Glycyrrhizic Acid Ammonium Salt, and Boric Acid Nanoparticles against Phytoplasma Associated with Faba Bean

**DOI:** 10.3390/molecules27051467

**Published:** 2022-02-22

**Authors:** Eman A. Ahmed, Tahsin Shoala, Abdelsattar Abdelkhalik, Hoda A. S. El-Garhy, Ismail A. Ismail, Amro A. Farrag

**Affiliations:** 1 Virus and Phytoplasma Research Department, Plant Pathology Research Institute, Agricultural Research Center (ARC), Giza 12619, Egypt; mnassar9977@gmail.com; 2 Environmental Biotechnology Department, College of Biotechnology, Misr University for Science and Technology, Giza 12563, Egypt; 3 Horticulture Department, Faculty of Agriculture, Fayoum University, Fayoum 63514, Egypt; aga04@fayoum.edu.eg; 4 Genetics and Genetic Engineering Department, Faculty of Agriculture, Benha University, Qalyubia 13736, Egypt; hoda.algarhy@fagr.bu.edu.eg; 5 Department of Biology, College of Science, Taif University, P.O. Box 11099, Taif 21944, Saudi Arabia; i.ismail@tu.edu.sa

**Keywords:** phytoplasma, faba bean, salicylic acid, glycyrrhizic acid ammonium salt, boric acid, nanoparticles, pods yield

## Abstract

Phytoplasmas are economically important plant pathogenic bacterial diseases, causing severe yield losses worldwide. In this study, we tested nanoformulations such as glycyrrhizic acid ammonium salt (GAS), salicylic acid (SA), and boric acid (BA) as novel antimicrobial agents inducing the resistance against the phytoplasma disease in faba bean. The nanoparticles (NP) were foliar-applied to naturally phytoplasma-infected faba bean with three concentrations from each of SA, GAS, and BA, under field conditions. Nested PCR (using universal primer pairs P1/P7 and R16F2n/R16R2) were reacted positively with all symptomatic samples and gave a product size of approximately 1200 bp, while the healthy plant gave no results. Transmission electron microscopy examinations of phytoplasma-infected faba bean plants treated with different nanoparticles revealed that severe damage occurred in phytoplasma particle’s structure, degradation, malformation, lysis in the cell membrane, and the cytoplasmic leakage followed by complete lysis of phytoplasma cells. Exogenous application of GAS-NP (1.68 µM), SA-NP (0.28 µM), and BA-NP (0.124 µM) suppressed the infection percentage of phytoplasma by 75%, 50%, and 20%, and the disease severity by 84%, 64%, and 54%, respectively. Foliar application of nanoparticles improved Fv/Fm (maximum quantum efficiency of PSII Photochemistry), PI (the performance index), SPAD chlorophyll (the relative chlorophyll content), shoots height, and leaves number, thus inducing recovery of the plant biomass and green pods yield. The most effective treatment was GAS-NP at 1.68 µM that mediated substantial increases in the shoots’ fresh weight, shoots’ dry weight, number of pods per plant, and green pods yield by 230%, 244%, 202% and 178%, respectively, compared to those of infected plants not sprayed with nanoparticles. This study demonstrated the utility of using nanoparticles, particularly GAS-NP at 1.68 µM to suppress the phytoplasma infection.

## 1. Introduction

The faba bean (*Vicia faba* L.) is a major food and feed grain legume for both humans and animals [1]. Because of their nutritious value, both fresh pods and dry seeds are consumed worldwide [2]. Mature faba bean seeds are high in protein (up to 25% in dry seeds), carbohydrates, cellulose, vitamin C, and minerals [2,3].

Various plant diseases (e.g., fungal, bacterial, viral, etc.) have been documented to infect this crop. Phytoplasma-related disease is considered as one of the most severe in yield-reduction worldwide [4]. Phytoplasma refers to microscopic, phytopathogenic, cell wall-less prokaryotes of the Mollicutes class that were previously designated as mycoplasma-like organisms. They are obligate parasites that inhabit host plant phloem tissue and insect host hemolymph. They are persistently transferred to other plants by phloem-sucking insects such as leafhoppers, plant hoppers, and psyllids [5]. Phytoplasma has been shown to be associated with the faba bean. The most common symptoms of phytoplasma infections of the faba bean are phyllody, shortening of internodes, flower virescence, witches’ broom, small leaves, and yellowing symptoms [6,7,8,9,10].

Control of phytoplasma is commonly accomplished by a variety of management techniques, including vector control, removal of diseased plants, and elimination of pathogens from infected plants using meristem tip culture, antibiotics, or other chemicals [11,12]. Furthermore, several uses of resistance inducer chemicals play an important role in phytoplasma control. Resistance inducers are chemical substances that allow the plant defense system to inhibit infections [12]. However, no single effective control technique has been established thus far. Researchers are trying to focus on sustainable approaches to suppress phytoplasmic disease development in plants. Exogenous elicitors are thought to be a promising strategy for developing plant resistance to phytoplasma infection.

Salicylic acid (SA) is a naturally present phenolic compound found in plants that plays a critical role in the signal transduction pathways that initiate systemic acquired resistance (SAR). SA regulates seed germination, respiration, photosynthesis, vegetative growth, flower formation, senescence, thermogenesis, and cellular redox homeostasis in plants [13,14,15,16,17,18]. External application of SA to various areas of the plant activates numerous metabolic pathways and can be used as an alternate method of treating illnesses and boosting plant output. The bioactivity of SA used externally in plants can be influenced by a variety of parameters, including concentration, treatment time, plant age, species, and target regions [17]. SA has a dual role in plant protection, leading to distinct resistance pathways and having a beneficial effect on phytopathogens.

Glycyrrhizic acid (GA) is a key bioactive substance in licorice that is obtained from the rhizomes and roots of *Glycyrrhiza glabra*. Licorice has been widely used as a herbal medicine to treat a variety of diseases for its antiviral [19], antibacterial [20,21], antifungal [22], antioxidant [23], antitumor [24,25], antiulcer [26], and anti-inflammatory effects.

Boric acid (H_3_BO_3_; BA) is a boron chemical with antibacterial effects. This chemical has been employed as a preservative in a variety of industries, including timber, bagasse, and food items, as well as for medical applications [27]. Because of its antibacterial characteristics, boric acid has been used as a pesticide to kill a variety of pests, including fleas and cockroaches. Getting rid of fungus and insects (such as termites) [28,29]. As a result, boric acid is thought to be a potential therapy for a variety of infectious illnesses [27].

The progress of technologies such as nanotechnology has opened new scopes for the treatment of many plant diseases, including viruses [30,31]. Engineered nanoparticles (NPs) are increasingly being used as bactericides/fungicides and as nanofertilizers in disease management techniques to enhance plant health. They can be utilized as protectants or for specific and targeted administration of an active compound, such as a pesticide, through adsorption, encapsulation, and/or conjugation. The potential for a new generation of insecticides and other actives for plant disease management will grow as agricultural nanotechnology advances [31]. In this context, the application of different bioactive chemical compounds in the form of nanoparticles (NPs) has attracted attention because of the multiple advantages that are presented, such as low manufacturing cost, the delivery of drugs to specific sites, less invasive therapies, and greater efficiency in treatment and recovery.

Most importantly, different reports indicated the suppression effect and a favorable control impact on plant viruses and phytoplasma infection; however, the effect might vary depending on nanoparticle concentration [12,31,32].

Transmission electron microscopy (TEM) has been utilized to characterize the morphology of phytoplasmas in plant host phloem tissue [33,34]. They appear in the sieve elements (SEs) of phloem tissue as spherical to oval or polymorphic cells with a diameter of 200–800 nm [35,36]. Due to the difficulty of cultivating phytoplasmas in vitro, DNA-based techniques for identifying and classifying phytoplasmas have been developed. The polymerase chain reaction (PCR) amplifies the 16S rRNA gene, which is ubiquitous across prokaryotes due to its conserved and varied sections, which enhances the detection and identification of phytoplasmas [37,38,39,40]. Therefore, nanoparticles under study could be a novel and promising elicitor to fight off phytoplasma diseases, as there are some similarities with bacteria.

The purpose of this study was (i) the molecular detection of phytoplasma associated with faba bean, (ii) studying the inhibitory effects of the SA GAS and BA nanoparticles at various doses on phytoplasma infection under field conditions, and (iii) evaluating the efficiency of these treatments for controlling phytoplasma through determination of the effects of the treatments on phytoplasma cell’s structure, photosynthetic efficiency, growth, and yield of faba bean plants.

## 2. Materials and Methods

### 2.1. Chemical Synthesis of Nanomaterials

Glycyrrhizic acid ammonium salt (CAS number: 53956-04-0), salicylic acid (CAS number: 20283-92-5), and boric acid (CAS Number: 1405-86-3) were purchased from Sigma-Aldrich (St. Louis, MO, USA). To prepare GAS-NPs, SA-NPs, and BA-NPs, 10 mg of GAS, 10 mg of SA, and 0.2 mg of BA were dissolved in 10, 10, and 1 mL of absolute ethanol, respectively, and sonicated for an hour at room temperature (20–25 °C) using ultrasonic power and frequency of 50 kHz (XUBA3Analogue Ul-ta-sonic Bath, Grant Company, Saint Joseph, MO, USA).

### 2.2. Characterization of Nanomaterials by Using Dynamic Light Scattering (DLS)

The distribution and the particle size of GAS-NPs, SA-NPs, and BA-NPs were determined using a Zetasizer Nano ZS and a dynamic light scattering method (Malvern Instruments, Malvern, UK). Before measurement, 30 µL of the produced nanoparticles was diluted in 3 mL of deionized water under room temperature. The particle size was calculated using the mean of the Z-averages of three different batches of each nanoparticles type mentioned above.

### 2.3. Transmission Electron Microscopy (TEM)

Transmission electron microscopy was used to characterize the nanomaterials’ morphology of all the three nanoparticle types. In this regard, a drop of the nanoparticles solution was sonicated and then put on carbon-coated copper grids (CCG) and totally dried by allowing water to evaporate at room temperature. At the Regional Center for Mycology and Biotechnology (RCMB) of Al-Azhar University, electron micrographs were acquired using a JEOL GEM-1010 transmission electron microscope at 70 kV [41].

Additionally, TEM was used to characterize phytoplasma and study the effect of the nanomaterials on its structure. Examination of ultra-thin section from tissues of untreated naturally infected faba bean and healthy and infected plants treated with different nanoparticles were carried out in Electron Microscopy Lab, Faculty of Agriculture, Cairo University. In brief, the plant samples were firstly prepared, fixed in 2% (*v*/*v*) glutaraldehide dissolved in 0.1 M sodium cacodylate buffer (pH 7.2) for 2 h at 4 °C and fixed again in 1% (*v*/*v*) osmium tetroxide for 1.5 h. The samples dehydrated with ascending concentrations of ethanol for 15 min for each concentration as described by Ahmed et al. (2014). After dehydration, ultra-thin sections were cut using an ultra microtome Leica model EM-UC6 at thickness of 70 nm, mounted on copper grids (400 mish). Sections were stained with double stain (Uranyl acetate 2% for 10 min followed by 0.4% Lead citrate for 5 min and examined by transmission electron microscope (JOELJM-14100)). Images were captured by a CCD camera model AMT.

### 2.4. Experimental Design and Plant Material

Field experiment was carried out during successive growth season (2020) at the Experimental Research station of Faculty of Agriculture, Fayom University (29.2938° N; 30.9144° E), Fayom Governorate. This area was selected based on survey results conducted in 2019 of phytoplasma infection on different crops and in different regions of Fayoum governorate, Egypt, including our study area. Our results confirmed that this region was highly epidemic for phytoplasma infection on the faba bean (unpublished data). The experiment was designed in a complete randomized block design (CRBD) with three replicates, where the applied treatments were randomly distributed. Healthy seeds of *Vicia faba* L. (cv. Giza 40) (obtained from the Egyptian Agriculture Ministry) were used in this experiment. The seeds were first rinsed with distilled water before being sterilized with a solution of sodium hypochlorite (1%; *v*/*v*) for about two minutes. The seed surface was then cleaned from the sterilization solution with distilled water before being dried at room temperature. The seeds were sown on 21 October 2020 in hills with 10–20 × 65 cm of plant and row spacing.

**Figure 1:**
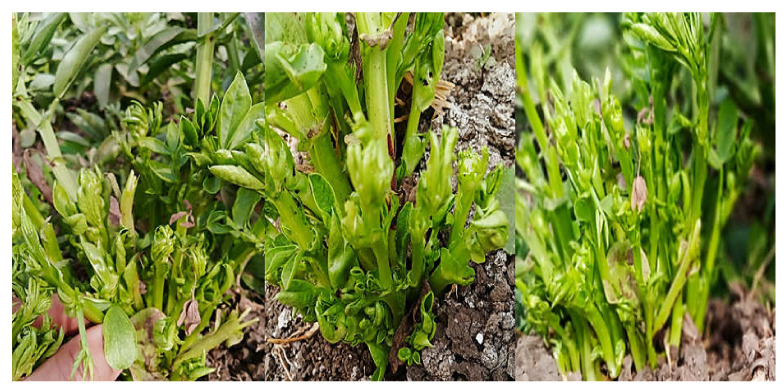
View of experimental field showing natural infection of faba bean associated with phytoplasma.

Leaf samples of faba bean were randomly collected from each experimental unit for detection of phytoplasma by nested PCR at the begining of phytoplasma-related symptom apperance on faba bean and before treatment application (Figure 1).

### 2.5. Treatments Application

Salicylic acid (SA-NPs), glycyrrhizic acid ammonium salt (GAS-NPs), and boric acid (BA-NPs) nanoparticles were foliar sprayed two times within three weeks with three concentrations as treatments at the beginning of phytoplasma-related symptom appearance as shown in Table 1. Experimental research design was divided into eleven treatments, first one prepared for natural phytoplasma-infected plants (positive control), from two to ten included foliar spraying of three concentrations from SA-NPs, GAS-NPs, and BA-NPs for naturally infected plants with phytoplasma, the eleventh treatment included healthy faba bean plants (negative control). To maintain healthy plants in the eleventh treatment without phytoplasma infection during season, the faba bean plants were foliar sprayed weekly with Actara^®^ 25% WG (Syngenta crop protection Inc., active substance thiamethoxam) at a concentration of 96 g 480^−1^ L ha^−1^ water up to flowering stage. The other treatments were left at the same time without any control to the insect vector for the occurrence of the natural infection by phytoplasma.

### 2.6. Nucleic Acid Extraction

A DNeasy Plant Pro Kit (Qiagen) was used to extract total DNA from untreated naturally infected faba bean, healthy, and infected plants treated with different nanoparticles.

### 2.7. Nested PCR for Phytoplasma Detection

Nested PCR was performed using the isolated DNA as a template. In the first PCR, a universal phytoplasma primer pair was designed according to Eppo bulletin PM7/133 [42]. First PCR was conducted using the primer pair P1/P7 to amplify an 1800 pb product and R16F2n/R16R2 to amplify 1200 pb in the second PCR. Both PCR reactions were performed in 25 μL containing 3 μL of extracted DNA, 1.5 μL of 10 pmol of each primer, 12.5 μL of amaR OnePCR™ (genedirex) and 6.5 μL of sterile water. The product of the first PCR was diluted 1:10 and used as a template for the second PCR round. The amplification-optimized PCR protocol started with a denaturation step at 94 °C for 3 min, followed by 40 cycles consisting of denaturation at 94 °C for 30 s, annealing at 53 °C for 30 s, extension at 72 °C for 1 min, and a final extension step was added for 10 min at 72 °C. The PCR products were analyzed using 1% Agarose Gel stained with EZ-View stain (Biomatik Kitchener, ON, Canada).

### 2.8. Incidence and Severity of the Phytoplasma Disease

**Figure 2:**
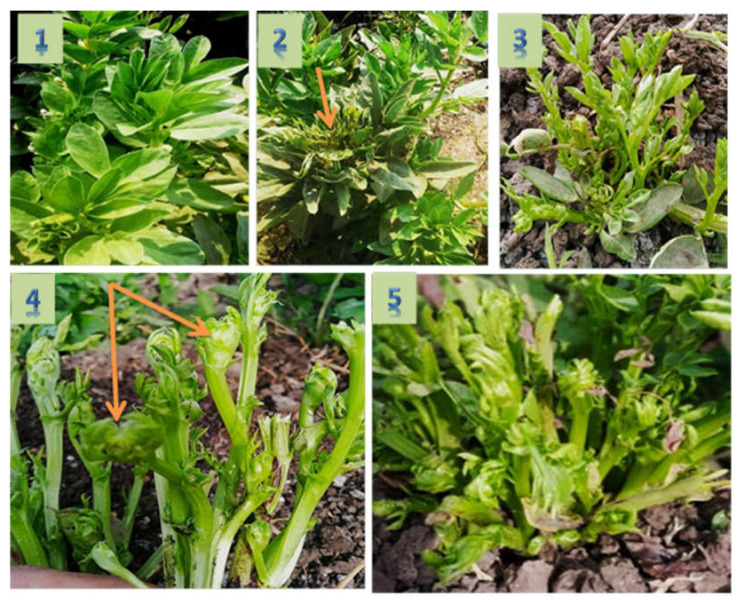
Disease severity index (DSI): index from 1 to 5, where 1 = no symptoms; 2 = yellow and little leaves; 3 = little leaves and stunting; 4 = shoot proliferation, yellowing, dwarfing; 5 = severe stunting and witches’ broom.

The disease incidence and severity of phytoplasma for all treatments were determined. The disease incidence percentage was estimated based on the visual symptoms developed on diseased plants using the formula,

D.I.% = *n*/*N* × 100,

where D.I. = disease incidence; *n* = number of infected plants, and *N* = the total number of plants assessed. The severity index of the disease described the damage caused by phytoplasma was recorded using disease severity index (DSI) (Figure 2). Percentage of disease severity was calculated using the following equation [43].

$$D.S.\%=\frac{\sum \left(\mathrm{Disease}\;\mathrm{grade}\;x\;\mathrm{No}\;\mathrm{of}\;\mathrm{plant}\;\mathrm{in}\;\mathrm{each}\;\mathrm{grade}\right)}{\mathrm{Total}\;\mathrm{no}\;\mathrm{of}\;\mathrm{tested}\;\mathrm{plants}\;x\;\mathrm{highest}\;\mathrm{disease}\;\mathrm{grade}}\times 100$$

### 2.9. Photosynthetic Efficiency, Growth, and Green Pods Yield

Faba bean photosynthetic efficiency and growth traits were analyzed at green pods harvesting. The chlorophyll fluorescence measurements were performed with a handy PEA fluorometer (Hansatech Instruments Ltd., Kings Lynn, UK). *F_v_*/*F_m_*; the maximum quantum yield of PSII (*Fv*/*Fm*) (maximum quantum efficiency of PSII photochemistry) was calculated using the equation: *F_v_*/*F_m_* = (*F_m_* – *F*_0_)/*F_m_* [44]. The performance index (PI) of photosynthesis quantifies electron flow rate, absorption, trapping, and dissipation of excitation energy, determined as depicted by [45]. Using a SPAD-502 chlorophyll meter (Minolta, Osaka, Japan), the relative chlorophyll content (SPAD chlorophyll) was measured. Five plants from each experimental plot were randomly taken to determine shoots height, number of leaves, and branches per plant. Thereafter, the leaves and branches (hereinafter called shoots) were weighed to record shoots fresh weight (g), after that they were placed in the oven and dried at 70 ± 2 °C until a constant weight was reached to record the shoots’ dry weight (g). All plants of each experimental plot were removed to determine the green pods yield per hectare, and the numbers of green pods per plant were counted.

### 2.10. Statistical Analysis

All data of disease incidence and severity, photosynthetic efficiency, growth traits, and pods yield were analyzed by analysis of variance (ANOVA) using Genstat XII (VSN International Ltd., Oxford, UK). Duncan’s multiple range tests at probability ≤0.05 were used as the mean separation test, and the data are presented as means ± standard error (S.E).

## 3. Results

### 3.1. Symptomatology and Nested PCR for Phytoplasma Detection

**Figure 3:**
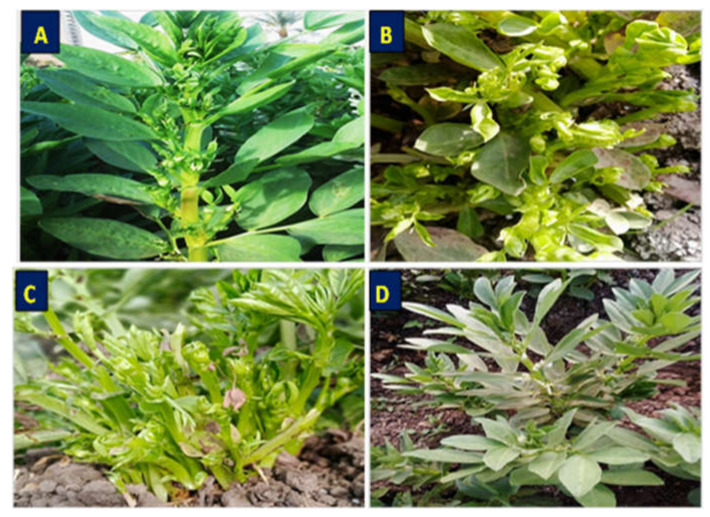
Naturally infected faba bean plants showing phytoplasma disease; (**A**) phyllody symptoms; (**B**) yellowing and little leaves; (**C**) shoot proliferation, witches’ broom, and stunting, and (**D**) asymptomatic faba bean plant.

Symptoms of phytoplasma appeared on naturally infected faba bean plants in the experimental field after 35–40 days from cultivation. These symptoms included phyllody (Figure 3A), yellowing and little leaves (Figure 3B), shoot proliferation, witches’ broom, and stunting (Figure 3C) when compared with healthy plants (Figure 3D). Nested PCR was performed using universal primer pairs P1/P7 and R16F2n/R16R2 for phytoplasma detection. All symptomatic samples showed PCR products with approximate size 1200 bp, while the healthy plants gave no PCR products. 

### 3.2. Characterization of Nanomaterials

**Figure 4:**
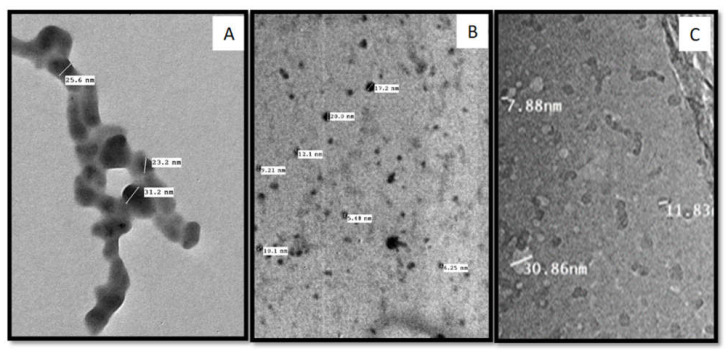
Transmission electron microscopy (TEM) image of prepared (**A**) GAS-NPs, (**B**) SA-NPs, and (**C**) BA-NPs.

TEM analyses showed that all the produced GAS-NPs, SA-NPs, and BA-NPs were poly-dispersed with an average size of <100 nm. The TEM micrograph showed that GAS-NPs were oval shaped with an average size ranged between 23.2 and 31.2 nm (Figure 4A). However, SA-NPs were produced in a circle shape with an average size ranged between 6.25 and 20 nm. (Figure 4B), and BA-NPs had an irregular shape with average size of 7.88–30.86 nm (Figure 4C).

### 3.3. Transmission Electron Microscopy (TEM)

**Figure 5:**
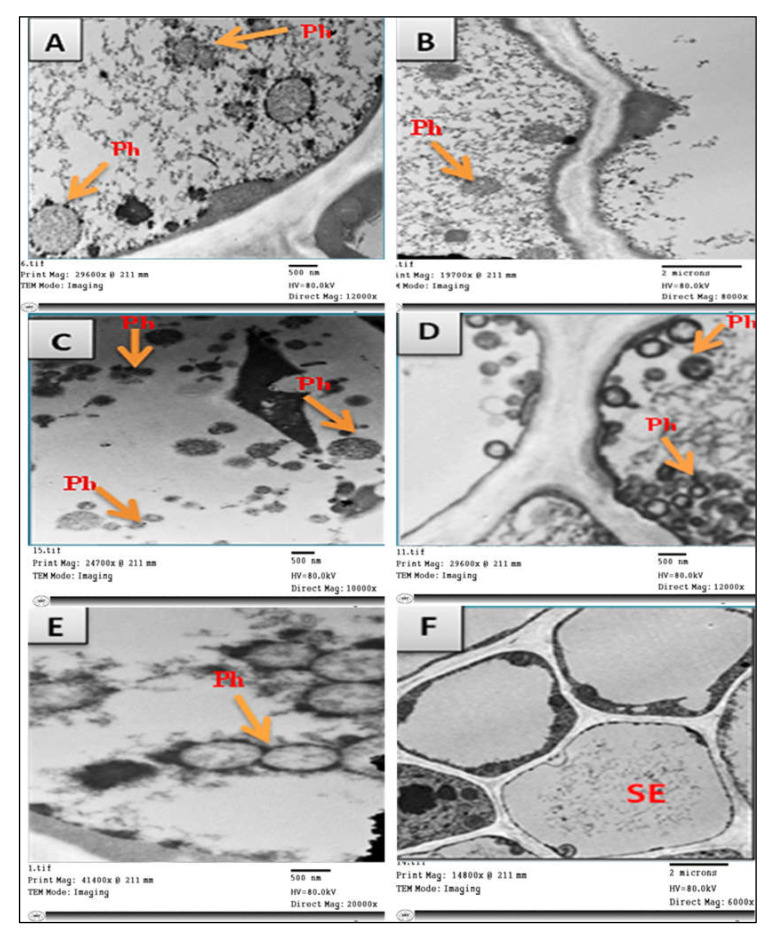
Transmission electron micrograph of sieve elements of phytoplasma-infected faba bean showing the effect of plants treated with boric acid (BA-NPs), salicylic acid (SA-NPs), and glycyrrhizic acid (GAS-NPs) nanoparticles on phytoplasma units at the best concentrations of 0.124, 0.28, and 1.68 µM (**A**–**C**), respectively, compared with phytoplasma controls (**D**,**E**) and healthy controls (**F**). Ph = Phytoplasma, SE = sieve element.

TEM was used for detection of phytoplasma and studying the effect of nanomaterials on phytoplasma structure. Ultra-thin sections of untreated phytoplasma-infected faba bean plants showed the presence of phytoplasma particles in groups arranged next to the cell membrane of infected sieve element cells (Figure 5D,E). These particles were mostly spherical or oblong in shape, bounded by a unit membrane with a diameter of 200–800 nm (Figure 5D,E). According to TEM, no phytoplasma particles were observed in the phloem tissues of healthy plants (Figure 5F). Treatments of phytoplasma-infected faba bean plants with different nanoparticles (GAS-NPs, SA-NPs, and BA-NPs) showed a significant direct effect on the phytoplasma structure. The treatment with BA-NPs caused structural changes in phytoplasma particles, partial degradation, and malformation in cell membrane (Figure 5A). However, foliar application of GAS-NPs and SA-NPs caused severe damage in the phytoplasma particle structure, detachment of cytoplasm from the cell membrane, degradation and lysis in the cell membrane, and cytoplasmic leakage followed by complete lysis of phytoplasma cells, resulting in the disruption of cellular metabolism (Figure 5B,C).

### 3.4. Effect of SA-NP, GAS-NP, and BA-NP Treatments on Phytoplasma-Infected Faba Bean Plants

**Figure 6:**
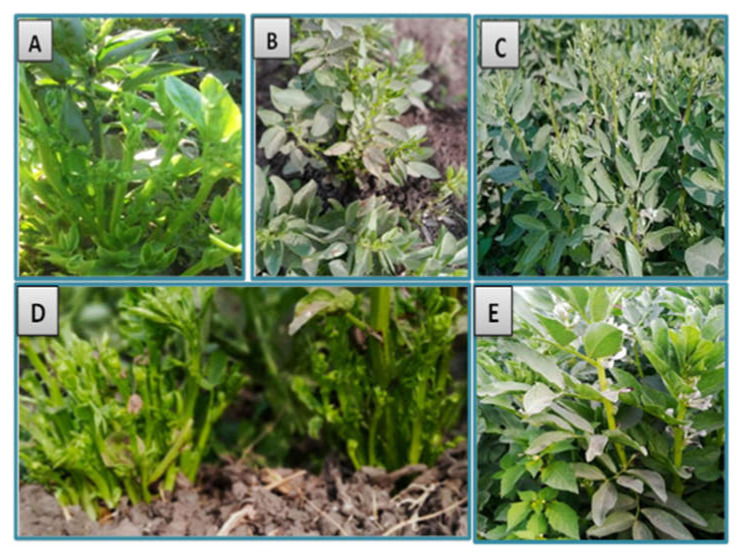
Effect of treatments with boric acid (BA-NPs), salicylic acid (SA-NPs), and glycyrrhizic acid (GA-NPs) nanoparticles on symptoms of phytoplasma-infected faba bean plants at the best concentrations of 0.124, 0.28, and 1.68 µM (**A**–**C**), respectively, compared with phytoplasma-positive control (**D**) and healthy control (**E**).

Data presented in the table indicated that all different treatments with nanomaterials reduced the percentage of infected plants with phytoplasma. Application of GAS-NPs at 1.68 µM showed the most effective treatment with great reduction in the percentage of infection, reaching 75%, followed by 50% and 20% for SA-NPs at 0.28 µM and BA-NP at 0.124 µM, respectively, when compared with that in untreated infected plants. Concerning disease severity, nanoparticle treatments displayed different degree effects on phytoplasma symptom development, ranging from hidden to mild. GAS-NP treatment led to the disappearance of phytoplasma symptoms (Figure 6C) as well as decreased disease severity by 84.25% in comparison with that in untreated phytoplasma-infected plants (positive control) (Figure 6D). The infected plants reacted with SA-NP at 0.28 µM and gave mild systemic symptoms (Figure 6B), followed by reduction in disease severity by 63.7%. However, BA-NP had lower effects on reducing the symptoms of phytoplasma and disease severity (Figure 6A). Nested PCR results of all treated plants confirmed the presence of phytoplasma in spite of the reduction of phytoplasma symptoms 

**Figure 7:**
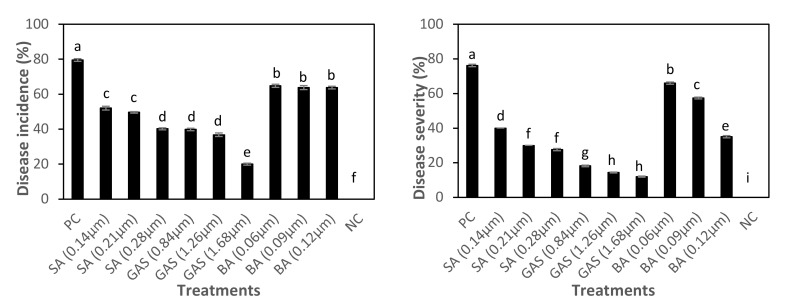
Variation in disease incidence and severity in negative control plants (NC), positive control plants (PC), and in response to foliage application of nano-salicylic acid (SA; 0.14, 0.21, and 0.28 μM), nano-glycyrrhizic acid ammonium salt (GAS; 0.84, 1.26, and 1.68 μM), and nano-boric acid (BA; 0.06, 0.09, and 0.12 μM) to phytoplasma-infected faba bean plants. Means followed by the same letter in each column were not significantly different according to Duncan’s multiple range test (*p* ≤ 0.05).

Results in Table 2 and Figure 7 showed that the percentage of disease severity decreased significantly to 12% in response to foliar application with 1.68 µM GAS-NPs compared to 76.2% in the positive control. Foliar treatments with different concentrations of GAS-NPs showed significant decrease in disease severity compared to that with other treatments with SA-NPs, BA-NPs, and positive control. The percentage of disease incidence recorded 20% in the exogenous application of GAS-NPs with 1.68 µM compared to 79.6% in the positive control, while the higher concentration of SA-NPs and BA-NPs showed 40.2% and 63.8%, respectively, of disease incidence.

### 3.5. Effect of SA-NP, GAS-NP, and BA-NP Treatments on Photosynthetic Efficiency, Growth, and Green Pods Yield of Phytoplasma-Infected Faba Bean Plants

**Figure 8:**
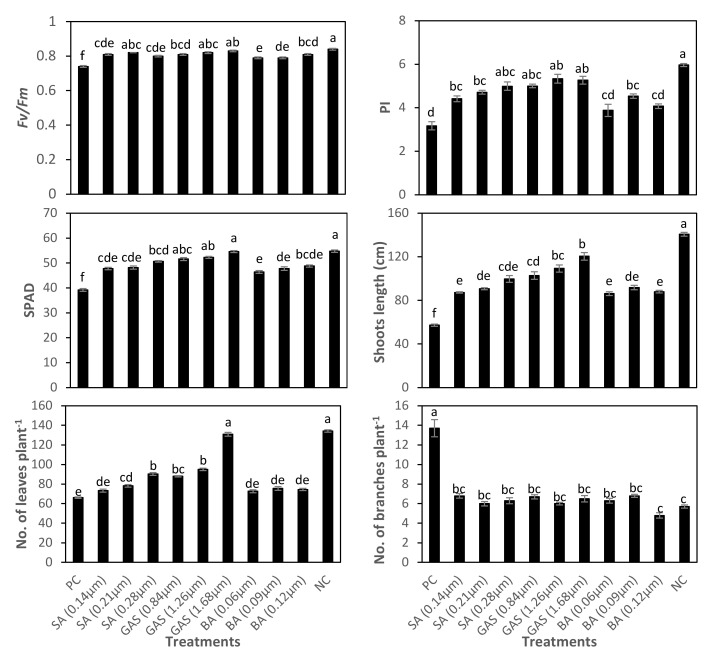
Variation in the photosynthetic efficiency (*F_v_*/*F_m_* and PI), SPAD chlorophyll, shoots length, leaves number, and number of branches in negative control plants (NC), positive control plants (PC), and in response to foliage application of nano-salicylic acid (SA; 0.14, 0.21, and 0.28 μM), nano-glycyrrhizic acid ammonium salt (GAS; 0.84, 1.26, and 1.68 μM), and nano-boric acid (BA; 0.06, 0.09, and 0.12 μM) to phytoplasma-infected faba bean plants. Means followed by the same letter in each column were not significantly different according to Duncan’s multiple range test (*p* ≤ 0.05).

Results in Table 3 and Figure 8 showed that the photosynthetic efficiency of PSII (*F_v_*/*F_m_* and PI), SPAD chlorophyll and growth traits were significantly affected by phytoplasma infection and foliar application of SA-NP, GAS-NP, and BA-NP. Infected faba beans with phytoplasma showed adverse effects on faba bean plants and recorded lower *F_v_*/*F_m_* by 11%, PI by 47%, SPAD chlorophyll by 28%, shoots height by 59%, and leaves number by 51%, while increasing number of branches by 141%, considered as normal symptoms for infected plants, in comparison to those in the healthy plants (NC). However, foliar application of GAS-NP and, to a lesser extent, SA-NP and BA-NP, improved these parameters of infected plants, highlighting that foliar application of GAS-NP at 1.68 µM to infected plants increased these traits by 12%, 66%, 39%, 110%, and 98%, respectively, while the number of branches decreased by 53%, compared with those in untreated phytoplasma-infected plants (PC) (Table 3, Figure 8).

**Figure 9:**
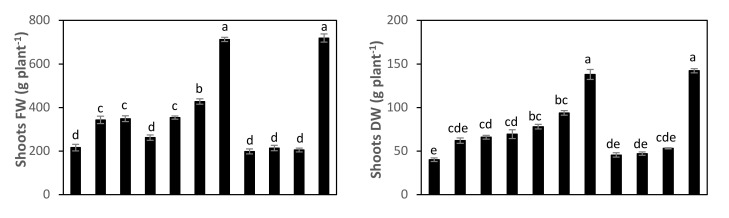

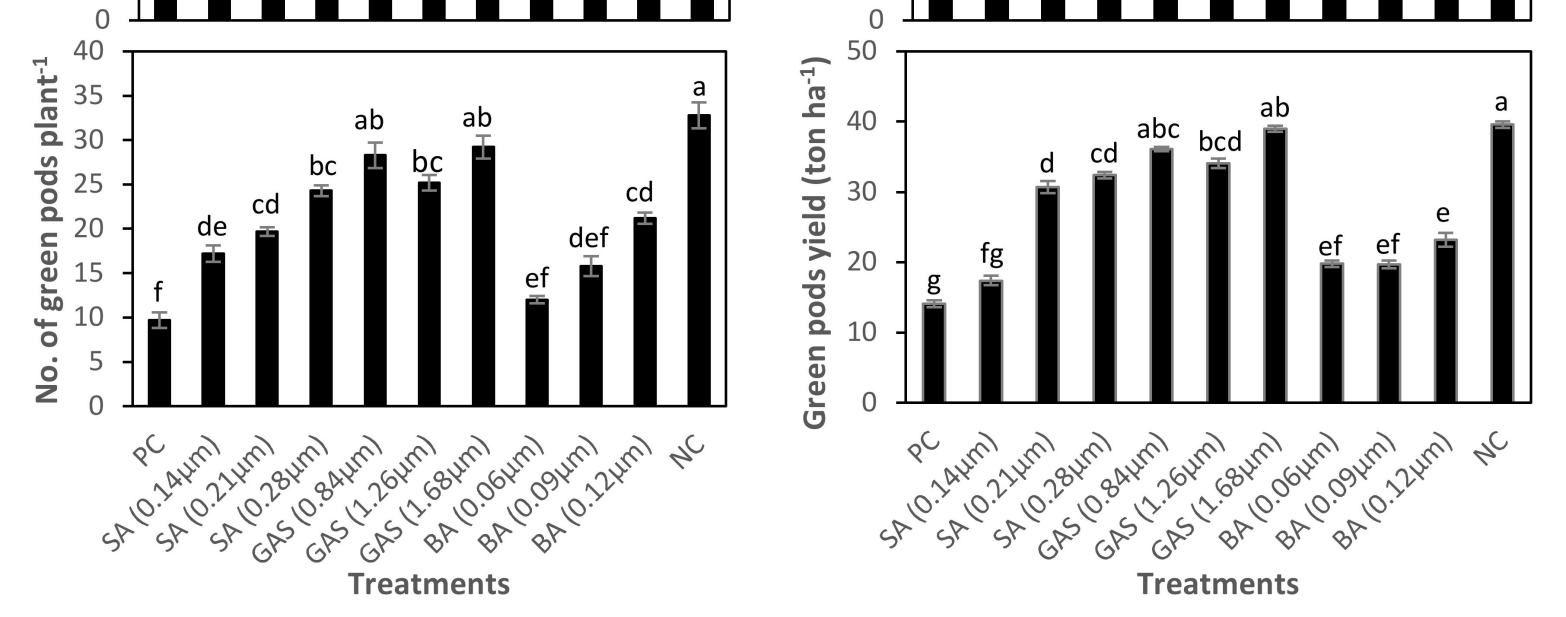
Variation in shoots fresh (FW) and dry weight (DW), number of pods, and green pods yield in negative control plants (NC), positive control plants (PC), and in response to foliage application of nano-salicylic acid (SA; 0.14, 0.21, and 0.28 μM), nano-glycyrrhizic acid ammonium salt (GAS; 0.84, 1.26, and 1.68 μM), and nano-boric acid (BA; 0.06, 0.09, and 0.12 μM) to phytoplasma-infected faba bean plants. Means followed by the same letter in each column were not significantly different according to Duncan’s multiple range test (*p* ≤ 0.05).

As shown in Table 4 and Figure 9, the phytoplasma infection caused considerable reductions in faba bean shoots fresh weight by 70%, shoots dry weight by 72%, number of green pods plant by 71%, and green pods yield by 65% compared with those of uninfected plants (NC). All foliar-applied nanoparticles particularly GAS-NP alleviated the negative effects of phytoplasma infection on shoots biomass and green pods yield of *Vicia faba* plants. Under phytoplasma infection, exogenous application of GAS-NP at 1.68 µM induced recovery of the biomass and yield reduction occurred through inducing significant increases in the shoots fresh weight (by 230%), shoots dry weight (by 244%), number of pods per plant (by 202%) and green pods yield (by 178%) compared to infected plant non-sprayed with nanoparticles (PC), and recorded similar values to the healthy plants (NC) (Table 4, Figure 9).

## 4. Discussion

Phytoplasma has been shown to be associated with the faba bean. Our results revealed that naturally infected faba bean plants associated with phytoplasma showed shoot proliferation, witches’ broom, stunting, phyllody, and yellowing and little leaves. Our plants had the usual phytoplasma infection symptoms. The greatest present management approaches to treat infected faba bean plants is eradicating infected plants using a safe quarantine protocol. Alternatively, nanotechnology has different applicable approaches against different phytopathogens. Our research was aiming to study the impact of glycyrrhizic acid ammonium salt (GAS-NPs), salicylic acid (SA-NPs), and boric acid (BA-NPs) nanoparticles against infected faba beans with phytoplasma under field conditions.

Exogenous treatments with different nanomaterials suppressed the percentage and severity of phytoplasma-infected plants. Additionally, spraying infected plants with nanomaterials enhanced the recovery speed process compared to that of untreated phytoplasma-infected plants. All nanoparticles, especially GAS-NPs, improved photosynthetic efficiency and growth parameters such as shoots height, leaves number, and number of branches of phytoplasma-infected plants compared with those of untreated phytoplasma-infected plants (PC).

Moreover, foliar application of nanoparticles led to significant increases in the shoots fresh weight, shoots dry weight, number of pods per plant, and green pods yield compared to those of phytoplasma-infected plants. The positive impacts of nanoparticles on infected plants may be due to their direct impact on the phytoplasma cell membrane. Foliar application of GAS-NPs and SA-NPs caused severe damage in the structure of phytoplasma’s cell membrane, detachment of cytoplasm from the cell membrane followed by lysis of cell membrane, and leakage of the cytoplasm. Consequently, disruption of cellular metabolism occurred and, finally, phytoplasma cells were completely lysed. Similar observations were previously reported in [46,47], where it was reported that nanomaterials caused damage to the bacterial cell wall and membranes through binding to the cell membrane, resulting in change of the membrane potential, with membrane depolarization leading to imbalance in metabolism processes. Boric acid nanoparticles enhanced the resistance of infected faba bean plants with phytoplasma, but not as greatly as the other two nanoparticles did.

Our research suggested that the following events could have happened according to the proposed hypothetical schematic diagram. Spraying the infected plants with nanoparticles might promote kinase 3 genes due to the induction of many signaling channels as well as the oxidative inducible gene (oxi1 gene), ROS, and calcium signaling cascades [48]. Reactive oxygen species (ROS) are automatically generated within a few seconds in response to both biotic and abiotic stressors; they may also act as protectants against both biotic and abiotic stresses [49]. ROS play an important role in activating Mitogen-activated protein kinases (MAPKs) genes, which are considered as well-studied signaling families in higher plants. Furthermore, MAPKs regulate a broad range of critical cellular functions, such as cell division, stress responses, metabolism, and many developmental processes [48]. Enlarging or decreasing leaf area or root length may occur because of the stress response, so that interruption of different stresses using nanoparticles might suppress different stressors and increase the recovery process. Oxidative signal Inducible gene 1 (Oxi1) is a necessary serine/threonine kinase that involves ROS accumulation to enhance plant tolerance to various stimuli and oxidative burst-mediated signaling in plant roots. Induction of Oxidative signal Inducible gene may enhance plant resistance and speed the recovery process, but upregulation may cause cell death as a quick response to the external stress [48]. According to the hypothetical schematic diagram, our study suggested that using GAS-NPs, SA-NPs, and BA-NPs triggered MAPK cascades and improved plant growth to some extent by increasing photosynthetic pathways’ growth hormones. Accordingly, leaf area, root length, nutrient materials, and water absorption may have been enhanced.

Nanotechnology may lead the management strategies against different phytopathogen to higher levels. Natural nanomaterials, which could be prepared and extracted from plants such as (GAS-NPs), play an important role in enhancing plant resistance against different stimuli without harming the environment [50]. Preparation of nanomaterials is a time and money-saving process. Nanomaterials could be stored at room temperature according to the prepared material, and their stability could allow them to last for a few months.

Nanotechnology may play a dual role in plant resistance against phytopathogens by interacting with phytopathogens and enhancing the growth pathways which cause a positive impact in plant resistance.

## 5. Conclusions

Nanotechnology could introduce a magic solution to control or manage phytopathogens. Natural nanoparticles such as GAS-NPs presented a great impact on plants infected with phytoplasma, enhancing the recovery stage and reducing the crop loss of faba beans. Application of natural products in nanoform could control or manage phytopathogens. Further research studies are required to cover this area.

## Figures and Tables

**Table 1:** Definition of treatments relative to applying SA-NP, GAS-NP, and BA-NP treatments on phytoplasma infection.

No. of Treatments	Treatments	Concentration (µM)
1	PC	Un treated
2	PIP + SA-NP	0.14
3	PIP + SA-NP	0.21
4	PIP + SA-NP	0.28
5	PIP + GAS-NP	0.84
6	PIP + GAS-NP	1.26
7	PIP + GAS-NP	1.68
8	PIP + BA-NP	0.06
9	PIP + BA-NP	0.09
10	PIP + BA-NP	0.124
11	NC	Untreated

PC = positive control (untreated phytoplasma-infected plants); SA-NP = salicylic acid nanoparticle; GAS-NP = glycyrrhizic acid ammonium salt nanoparticle; BA-NP = boric acid nanoparticle; PIP = phytoplasma-infected plants; NC = negative control (healthy plants).

**Table 2:** Effect of SA-NPs, GAS-NPs, and BA-NPs treatments on phytoplasma.

Treatments	Concentrations	Disease Incidence %	Disease Severity %
PC	---------	79.6 ± 1.5 ^a^	76.2 ± 1.5 ^a^
PIP + SA-NP_1_	0.14 µM	52.0 ± 2.2 ^c^	40.0 ± 1.0 ^d^
PIP + SA-NP_1.5_	0.21 µM	49.6 ± 0.9 ^c^	30.0 ± 1.0 ^f^
PIP + SA-NP_2_	0.28 µM	40.2 ± 1.1 ^d^	27.6 ± 1.2 ^f^
PIP + GAS-NP_1_	0.84 µM	39.8 ± 1.4 ^d^	18.2 ± 0.9 ^g^
PIP + GAS-NP_1.5_	1.26 µM	36.8 ± 2.0 ^d^	14.4 ± 0.5 ^h^
PIP + GAS-NP_2_	1.68 µM	20.0 ± 1.1 ^e^	12.0 ± 0.7 ^h^
PIP + BA-NP_1_	0.06 µM	64.8 ± 1.8 ^b^	66.0 ± 1.3 ^b^
PIP + BA-NP_1.5_	0.09 µM	63.8 ± 2.4 ^b^	57.4 ± 1.0 ^c^
PIP + BA-NP_2_	0.124 µM	63.8 ± 1.5 ^b^	35.0 ± 1.3 ^e^
NC	--------	0.0 ± 00 ^f^	0.0 ± 0.0 ^i^

Values refer to means ± standard error. Means followed by the same letter in each column were not significantly different according to Duncan’s multiple range test (*p* ≤ 0.05).

**Table 3:** Effects of foliar-applied salicylic acid (SA-NP), glycyrrhizic acid (GAS-NP), and boric acid (BA-NP) nanoparticles on the photosynthetic efficiency and growth parameters of phytoplasma-infected faba bean plants.

Treatments	*F_v_*/*F_m_*	PI	SPAD Chlorophyll	Shoots Height (cm)	No. of Leaves Plant^−1^	No. of Branches Plant^−1^
**PC**	0.74 ± 0.01 ^f^	3.17 ± 0.38 ^d^	39.2 ± 1.14 ^f^	57.3 ± 2.03 ^f^	66.2 ± 1.38 ^e^	13.7 ± 1.76 ^a^
**PIP+SA-NP_1_**	0.81 ± 0.01 ^cde^	4.41 ± 0.26 ^bc^	47.8 ± 0.95 ^cde^	87.3 ± 1.31 ^e^	73.2 ± 3.05 ^de^	6.8 ± 0.48 ^bc^
**PIP+SA-NP_1.5_**	0.82 ± 0.00 ^abc^	4.71 ± 0.17 ^bc^	48.2 ± 1.27 ^cde^	90.7 ± 1.75 ^de^	78.2 ± 2.80 ^cd^	6.0 ± 0.45 ^bc^
**PIP+SA-NP_2_**	0.80 ± 0.01 ^cde^	4.99 ± 0.39 ^abc^	50.7 ± 0.62 ^bcd^	99.7 ± 6.14 ^cde^	90.3 ± 2.91 ^b^	6.3 ± 0.61 ^bc^
**PIP+GAS-NP_1_**	0.81 ± 0.01 ^bcd^	5.00 ± 0.16 ^abc^	51.6 ± 1.18 ^abc^	102.8 ± 7.07 ^cd^	88.0 ± 1.57 ^bc^	6.7 ± 0.42 ^bc^
**PIP+GAS-NP_1.5_**	0.82 ± 0.01 ^abc^	5.33 ± 0.40 ^ab^	52.3 ± 0.78 ^ab^	109.3 ± 6.57 ^bc^	95.0 ± 2.58 ^b^	6.0 ± 0.26 ^bc^
**PIP+GAS-NP_2_**	0.83 ± 0.01 ^ab^	5.26 ± 0.36 ^ab^	54.6 ± 0.85 ^a^	120.5 ± 6.98 ^b^	130.8 ± 3.88 ^a^	6.5 ± 0.67 ^bc^
**PIP+BA-NP_1_**	0.79 ± 0.01 ^e^	3.88 ± 0.56 ^cd^	46.4 ± 1.14 ^e^	86.3 ± 3.52 ^e^	72.7 ± 3.04 ^de^	6.3 ± 0.49 ^bc^
**PIP+BA-NP_1.5_**	0.79 ± 0.01 ^de^	4.53 ± 0.20 ^bc^	47.8 ± 1.49 ^de^	91.8 ± 4.00 ^de^	75.5 ± 3.85 ^de^	6.8 ± 0.31 ^bc^
**PIP+BA-NP_2_**	0.81 ± 0.01 ^bcd^	4.07 ± 0.21 ^cd^	48.8 ± 0.91 ^bcde^	88.0 ± 2.38 ^e^	74.5 ± 2.14 ^de^	4.8 ± 0.54 ^c^
**NC**	0.84 ± 0.01 ^a^	5.96 ± 0.14 ^a^	54.8 ± 0.95 ^a^	140.7 ± 3.48 ^a^	134.2 ± 2.36 ^a^	5.7 ± 0.33 ^c^

Values refer to means ± standard error. Means followed by the same letter in each column were not significantly different according to Duncan’s multiple range test (*p* ≤ 0.05).

**Table 4:** Effects of foliar-applied salicylic acid (SA-NP), glycyrrhizic acid (GAS-NP), and boric acid (BA-NP) nanoparticles on biomass and green pods yield of phytoplasma-infected faba bean plants.

Treatments	Shoots Fresh Weight (g Plant^−1^)	Shoots Dry Weight (g Plant^−1^)	No. of Green Pods Plant^−1^	Green Pods Yield (ton ha^−1^)
**PC**	216.1 ± 29.8 ^d^	40.1 ± 3.98 ^e^	9.7 ± 1.75 ^f^	14.1 ± 1.00 ^g^
**PIP+SA-NP_1_**	343.5 ± 33.4 ^c^	62.0 ± 6.19 ^cde^	17.2 ± 1.82 ^de^	17.4 ± 1.35 ^fg^
**PIP+SA-NP_1.5_**	348.6 ± 26.6 ^c^	66.0 ± 4.34 ^cd^	19.7 ± 0.96 ^cd^	30.7 ± 1.71 ^d^
**PIP+SA-NP_2_**	262.0 ± 24.5 ^d^	69.5 ± 10.18 ^cd^	24.3 ± 1.20 ^bc^	32.4 ± 0.91 ^cd^
**PIP+GAS-NP_1_**	354.2 ± 14.4 ^c^	78.1 ± 5.40 ^bc^	28.3 ± 2.87 ^ab^	36.1 ± 0.61 ^abc^
**PIP+GAS-NP_1.5_**	427.8 ± 25.1 ^b^	93.8 ± 5.25 ^b^	25.2 ± 1.78 ^bc^	34.1 ± 1.36 ^bcd^
**PIP+GAS-NP_2_**	712.7 ± 20.0 ^a^	138.0 ± 11.53 ^a^	29.2 ± 2.56 ^ab^	39.0 ± 0.91 ^ab^
**PIP+BA-NP_1_**	198.4 ± 22.5 ^d^	45.6 ± 4.99 ^de^	12.0 ± 0.82 ^ef^	19.8 ± 0.89 ^ef^
**PIP+BA-NP_1.5_**	214.3 ± 24.9 ^d^	47.1 ± 3.98 ^de^	15.8 ± 2.21 ^def^	19.7 ± 1.13 ^ef^
**PIP+BA-NP_2_**	205.2 ± 17.7 ^d^	53.2 ± 1.33 ^cde^	21.2 ± 1.30 ^cd^	23.2 ± 1.93 ^e^
**NC**	719.2 ± 36.7 ^a^	142.2 ± 4.90 ^a^	32.8 ± 2.94 ^a^	39.6 ± 0.93 ^a^

Values refer to means ± standard error. Means followed by the same letter in each column were not significantly different according to Duncan’s multiple range test (*p* ≤ 0.05).

## Data Availability

The data presented in this study are available upon request from the corresponding author. The data are not publicly available due to privacy concerns.

## Sample Availability

Samples of the compounds are available from the authors.
